# Viral Status and Efficacy of Immunotherapy in Hepatocellular Carcinoma: A Systematic Review With Meta-Analysis

**DOI:** 10.3389/fimmu.2021.733530

**Published:** 2021-09-29

**Authors:** Ziniu Ding, Zhaoru Dong, Zhiqiang Chen, Jianguo Hong, Lunjie Yan, Haichao Li, Shengyu Yao, Yuchuan Yan, Yafei Yang, Chuncheng Yang, Tao Li

**Affiliations:** ^1^ Department of General Surgery, Qilu Hospital, Shandong University, Jinan, China; ^2^ Department of Hepatobiliary Surgery, The Second Hospital of Shandong University, Jinan, China

**Keywords:** viral status, immune checkpoint inhibitor, tumor microenvironment, hepatocellular carcinoma, meta-analysis

## Abstract

**Background and Aim:**

Immune checkpoint inhibitors (ICIs) have been widely used in hepatocellular carcinoma (HCC), while only a subset of patients experience clinical benefit. We aimed to investigate the effects of viral etiology on response to ICIs in HCC and depict the tumor immune microenvironment (TIME) of virally infected and uninfected HCC.

**Methods:**

A systematic search was conducted in PubMed, Web of Science, Embase, and the Cochrane central register of controlled trials up to August 2021. Clinical trials reporting the efficacy of ICIs in HCC were eligible. Baseline characteristics including first author, year of publication, National Clinical Trials (NCT) registry number, study region, sample sizes, interventions, line of treatment, and viral status were extracted. Meta-analysis was conducted to generate combined odds ratios (ORs) with 95% confidence intervals (CI) based on random or fixed effect model, depending on heterogeneity. Tumor immune microenvironment was depicted using ESTIMATE and CIBERSORT algorithm.

**Results:**

Eight studies involving 1,520 patients were included. Combined data suggested that there was no significant difference of objective response rate (ORR) between virally infected HCC and non-viral HCC patients [OR = 1.03 (95% CI, 0.77–1.37; I^2^ = 30.9%, p_H_ = 0.152)]. Similarly, difference was not observed on ORR between HBV-HCC and HCV-HCC patients [OR = 0.74 (95% CI, 0.52–1.06; I^2^ = 7.4%, p_H_ = 0.374)]. The infiltration of immune cells in the tumor microenvironment did not differ by etiology except for M0 macrophages, M2 macrophages, regulatory T cells, naive B cells, follicular helper T cells, activated dendritic cells, activated mast cells, and plasma cells. Despite differences in infiltration observed in specific cell types, the immune score and stromal score were generally comparable among etiology groups.

**Conclusion:**

Viral etiology may not be considered as the selection criteria for patients receiving ICIs in HCC, and viral status has little impact on TIME remodeling during HCC tumorigenesis.

## Introduction

Hepatocellular carcinoma (HCC) is ranked as the sixth most frequent malignancy, and the third most common cause of cancer-related mortality worldwide (1). Hepatic resection remains the major treatment option for early-stage HCC patients with well-preserved liver function. Sorafenib, a multityrosine kinase inhibitor (mTKI) that targets serine–threonine kinases Raf-1 and B-Raf, vascular endothelial growth factor receptors (VEGFRs), and other kinases including platelet-derived growth factor receptor β (PDGFR-β), has been considered the mainstay of treatment for advanced hepatocellular carcinoma (aHCC) in the past decade (2, 3). The treatment landscape of aHCC has changed dramatically over the past few years with the advent of immune checkpoint inhibitors (ICIs). Combination of atezolizumab with bevacizumab is now considered the standard of care for aHCC patients as the first-line treatment (3). Currently, the combination of different ICIs or ICI-based combination with mTKIs or antiangiogenic monoclonal antibodies are under exploration and may further revolutionize the first-line treatment scenario (4, 5). On the other hand, even though dual VEGF/PD-L1 blockade nearly doubled the objective response rate (ORR) of ICI monotherapy, still more than half of the patients did not respond (6). Therefore, it is of utmost importance to identify the subsets of HCC that are most likely to benefit from immunotherapies.

Approximately 13% of new cancer cases worldwide are associated with infections (7). It appears that viral-associated carcinoma has distinct biological and clinical features compared with viral-independent tumors. Previous studies revealed that human papillomavirus (HPV)-positive head and neck squamous cell carcinoma (HNSCC) presents a higher response rate and more lymphocyte infiltration than HPV-negative HNSCC (8–10). The etiology of HCC varies substantially by geographic region. Globally, approximately 54% cases of HCC are attributed to chronic hepatitis B virus (HBV) infection, especially in Asia and sub-Saharan Africa (11), while chronic hepatitis C virus (HCV) infection is likely the predominant cause of HCC in Italy and Japan (1). Hepatitis viral infection disrupts normal signaling pathways; leads to sustained hepatic inflammation, fibrosis, and aberrant hepatocyte regeneration; and exerts complex biological effects on the tumor microenvironment (TME) (12). It is still controversial whether there is a difference in clinical response rate for ICIs between HBV- and HCV-associated HCC. Some reported that responses occurred regardless of HCC etiology, while some others demonstrated that clinical activity was observed predominantly in uninfected or HCV-infected cohorts (13, 14).

The efficacy of immunotherapies is attuned by multiple immunosuppressive signals within the TME (15). Therefore, there exists intense interest in uncovering the underlying mechanisms leading to the immunosuppressive milieu. With the widespread use of high-throughput omics data, it is now possible to take an in-depth view of the global gene expression pattern and its involvement in HCC tumorigenesis and progression (16). Computational algorithms, including CIBERSORT and ESTIMATE, have also been developed for assessments on abundance of infiltrated immune cells based on gene expression profiles (14, 15). Altogether, these techniques provided reliable and economical methods to depict detailed tumor immune microenvironment (TIME) landscape.

HCC remains an extremely lethal disease worldwide, with poor prognosis and limited therapeutic options for patients in advanced stage (17). The mechanisms by which chronic hepatitis induces HCC may differ by etiology, and it remains unclear whether HCC etiology may be considered as selection criteria for immunotherapy. To our knowledge, this is the first study that conducted a comprehensive analysis on the etiology of HCC and the efficacy of ICIs. The current study first conducted a meta-analysis to investigate effects of viral etiology on response to ICIs in HCC. The TIME of virally infected and uninfected HCC was further depicted using bioinformatic methods.

## Materials and Methods

We followed the Preferred Reporting Items for Systematic Reviews and Meta-analyses (PRISMA) statement guidelines to design, analyze, and report our meta-analytic findings (18).

### Data Source and Searching Strategy

Online databases including PubMed, Web of Science, Embase, and the Cochrane central register of controlled trials were systematically reviewed up to August 2020. Meeting abstracts from European Society for Medical Oncology (ESMO), American Society of Clinical Oncology (ASCO), and American Association of Cancer Research (AACR) from 2015 onwards were also reviewed. Related studies were identified using the following terms, including hepatocellular carcinoma, immune checkpoint inhibitors, nivolumab, pembrolizumab, atezolizumab, avelumab, camrelizumab, SHR-1210, and durvalumab. Detailed searching strategy is presented in **Supplementary Table S1**. Additional papers were identified by a manual search of the references from the eligible articles.

### Criteria for Inclusion and Exclusion

The inclusion criteria were as follows: (i) clinical trials including anti-programmed cell death-1 (anti-PD-1)/programmed death-ligand 1 (PD-L1) monotherapy or in combination with other monoclonal antibodies (e.g., anti-CTLA-4 antibodies, anti-VEGFR antibodies) or TKIs; (ii) articles in English and present the data for any of the efficacy outcomes including ORR, complete response (CR), partial response (PR), stable disease (SD), progression of disease (PD), disease control rate (DCR), progression-free survival (PFS), and overall survival (OS). We did not apply any restrictions on phase of study, line of treatment, treatment duration, or drug dosage. Studies were excluded if they were (i) studies on conditions other than HCC, (ii) studies including combination therapies other than above mentioned, (iii) sharing the same participants completely or partially, and (iv) preclinical studies or case reports.

### Data Extraction and Quality Assessment

Included trials were reviewed in detail, and data extraction were independently performed by two investigators (ZD and LY). Disagreements were resolved by discussion and consensus with a third author (ZD). The following information including first author, year of publication, National Clinical Trials (NCT) registry number, study region, sample sizes, interventions, line of treatment, and viral status were extracted. Quality of eligible studies were assessed using a 20-criterion quality appraisal checklist reported previously (19). Criteria regarding study design, demographic characteristics, intervention, follow-up and outcomes, competing interest, and sources of financial support were incorporated in the checklist.

### Bioinformatics Analysis

Gene expression data and corresponding clinicopathological information of 729 HCC samples (GSE9843, GSE78737, GSE107170, and GSE121248) were acquired from The Cancer Genome Atlas (TCGA) (https://portal.gdc.cancer.gov/repository) and Gene Expression Omnibus (GEO) (https://www.ncbi.nlm.nih.gov/geo) databases. Viral statuses in TCGA-LIHC cohort were extracted from the supplemental material from the TCGA-LIHC marker paper (20). The fragments per kilobase million (FPKM) values of TCGA-LIHC datasets were transformed into transcripts per kilobase million (TPM) as previous described, which was believed to be identical to those from microarrays (21). Single-sample gene-set enrichment analysis (ssGSEA) is a unique GSEA method used to calculate separate enrichment scores for each sample (22). The infiltration level of different immune cell types in HCC samples was quantified using ssGSEA based on 29 immune-associated gene sets reported previously (23). The immune score, stromal score, and ESTIMATE score of each single HCC sample were estimated using the ESTIMATE algorithm to validate the effect of ssGSEA immune clustering (24). CIBERSORT deconvolution algorithm was then utilized to precisely measure the fractions of 22 human immune cell subsets in HCC samples (25).

### Statistical Analysis

The association between viral status and response to ICIs in HCC was assessed by odd ratios (ORs) with an estimate of 95% CIs. A synthesized OR >1 suggests a higher response rate to ICIs. Heterogeneity was measured by the Cochran Q statistic and the I^2^ statistic [100% (Q − df)/Q]. The p-value for heterogeneity was represented as p_H_. Random-effects model will be utilized once the I^2^ >50% and reach statistical significance. Otherwise, the fixed-effects model was used to generate the pooled meta-analysis. In addition, Labbe’ plot and Galbraith plot were generated to visually evaluate the heterogeneity among studies. The influence of a single study on the overall meta-analysis estimate was investigated by *metainf* Stata command. Publication bias was assessed by inspecting the symmetry of the funnel plot and tested with Egger’s test. All the statistical analysis conducted in the current meta-analysis were performed by STATA version 12.0 (Stata Corporation, College Station, TX).

## Results

### Study Selection

**Figure 1:**
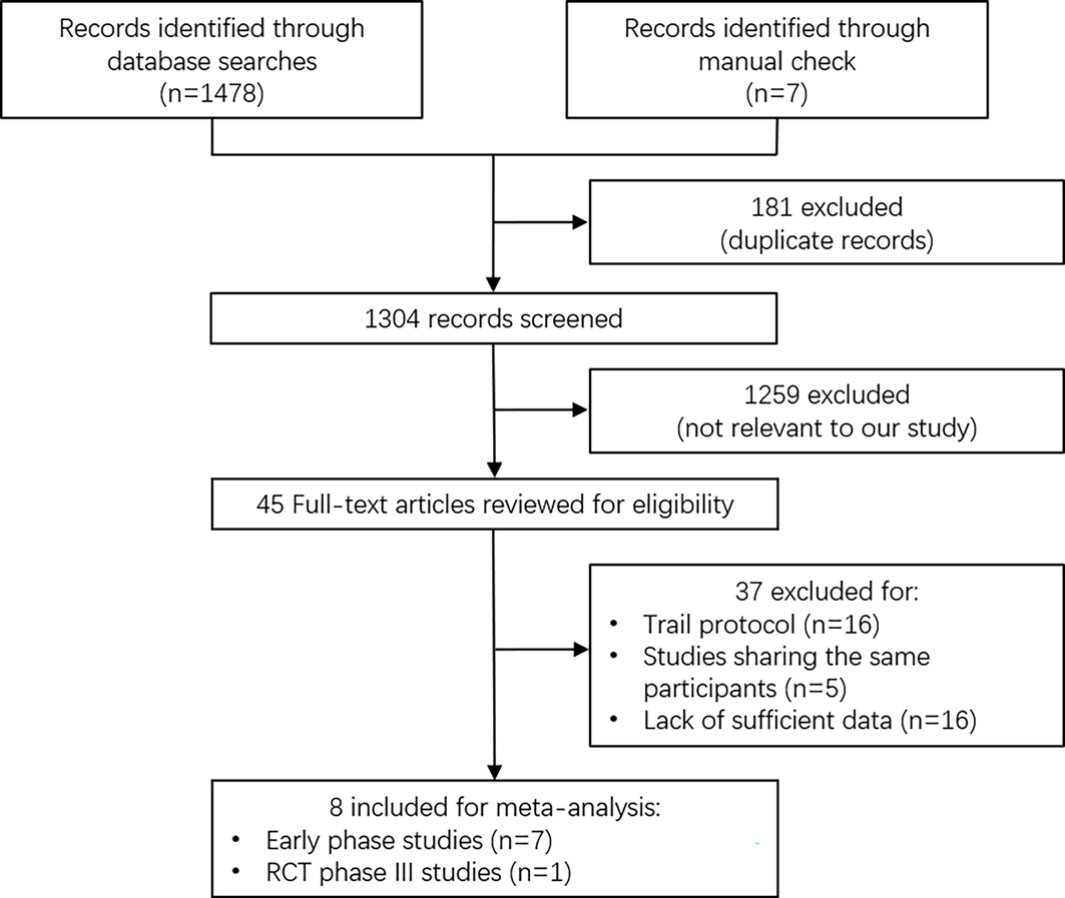
Flow diagram for study selection.

A total of 1,485 articles were retrieved based on the search terms given above, among which 181 duplicated records were removed. After initial screening, 1,259 articles not relevant to our study were removed. In total, 45 studies were eligible for a full-text assessment, of which 37 were removed, as they were trail protocol (n = 16), studies sharing the same participants (n = 5), or studies lack of sufficient data (n = 16). Finally, 8 studies with 1,520 patients were included for meta-analysis, among which 7 were early phase trails and 1 was phase III randomized controlled trial (RCT) (**Figure 1**) (6, 13, 14, 16, 26–29). These included studies were trails on anti-PD-1/L1 monotherapy (nivolumab, pembrolizumab, and durvalumab), anti-PD-1/L1 combined with anti-CTLA-4 (tremelimumab and ipilimumab), and anti-PD-1 in combination with anti-VEGFR therapy (atezolizumab plus bevacizumab) **Table 1**. In the study conducted by Kelly et al., patients were treated with different immunotherapy agents, and we considered each to be a single study arm and analyzed it accordingly (16).

**Table 1:** Basic characteristics of eligible studies.

Study	Year	Region	No. of Pts	Study Registration No.	Intervention	Line of Treatment	Phase of Study	HCC Etiology		
								HBV	HCV	Uninfected
El-Khoueiry et al. (26)	2017	Global	262	NCT01658878	Nivolumab	Second line	I/II	66	60	136
Kelley et al. (16)	2021	Global	332	NCT02519348	Durvalumab plus Tremelimumab	Second line	I/II	123	95	114
Yau et al. (13)^a^	2020	Global	148	NCT01658878	Nivolumab plus Ipilimumab	Second line	I/II	75	33	33
Feun et al. (14)	2019	USA	29	NCT02658019	Pembrolizumab	Second line	II	5	9	15
Lee et al. (27)	2020	Global	104	NCT02715531	Atezolizumab plus Bevacizumab	First line	Ib	51	31	22
Zhu et al. (28)	2018	Global	104	NCT02702414	Pembrolizumab	Second line	II	21	26	57
Finn et al. (6)	2020	Global	501	NCT03434379	Atezolizumab plus Bevacizumab	First line	III	240	108	153
Wainberg et al. (29)^b^	2017	Global	40	NCT01693562	Duralumab	Second line	I/II	9	8	21

a Seven patients overall were reported as having both HBV and HCV infection.

b One HBV-positive patient was non-response evaluable.

### Viral Status Has no Impact on Response to Immunotherapy

**Figure 2:**
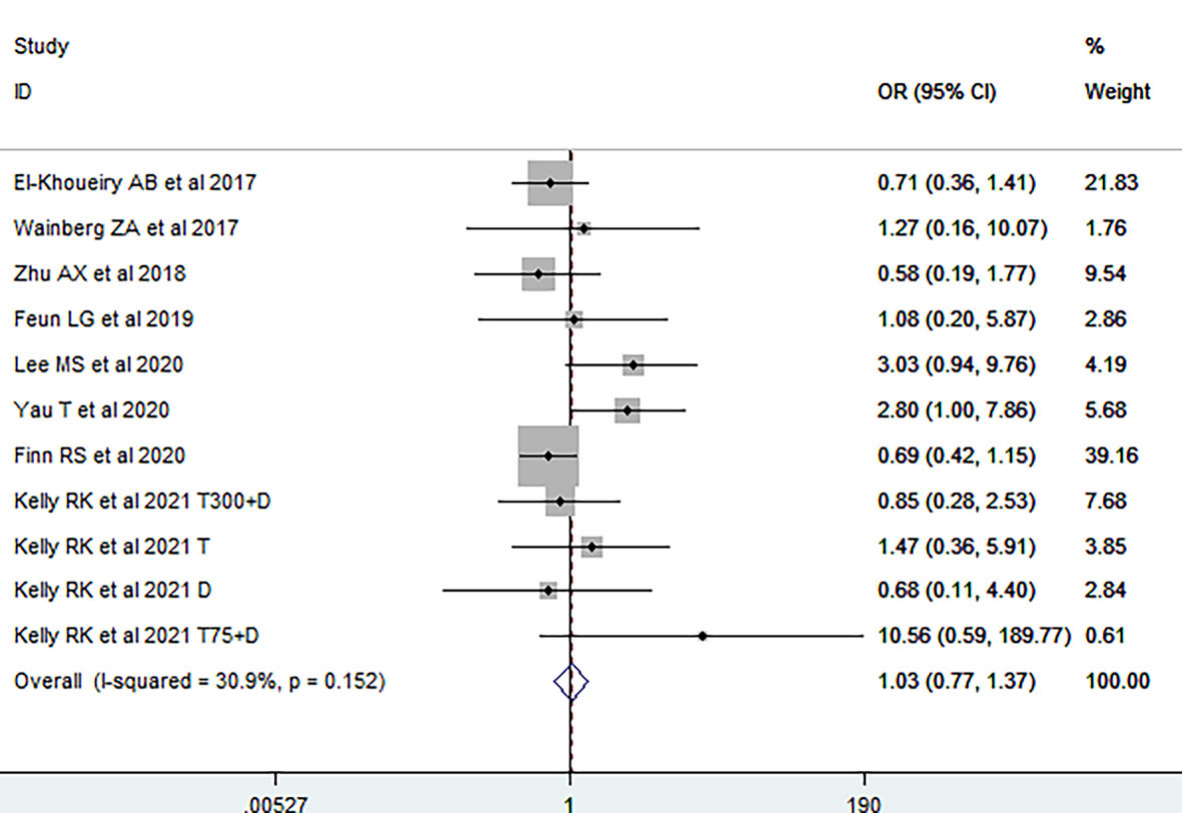
Pooled odds ratio of response rate between virally infected and uninfected HCC.

**Figure 3:**
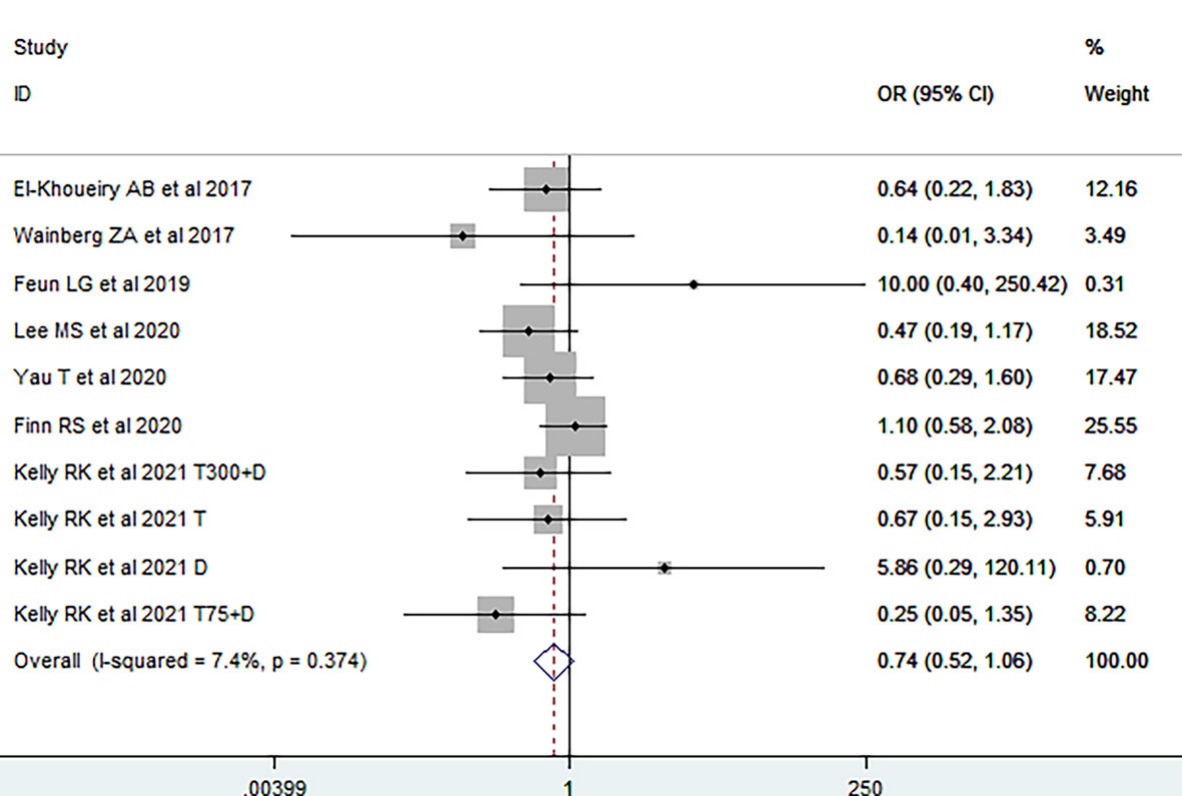
Forest plot of response rate in virally infected HCC stratified by etiology.

We first investigated the impact of viral etiology on the efficacy of immunotherapy; primary endpoint was ORR. It was demonstrated that no significant difference of ORR was observed between virally infected HCC and non-viral HCC patients [OR = 1.03 (95% CI, 0.77–1.37), **Figure 2**], without significant heterogeneity among studies (I^2^ = 30.9, p_H_ = 0.152). The funnel plot, sensitivity analysis, LAbbe’ plot, and Galbraith plot were further conducted, and no possible heterogeneity was observed (**Supplementary Figures S1A–D**). Fixed-effects meta-analysis was subsequently utilized to estimate the difference of ORR between HBV-HCC and HCV-HCC. The overall OR was 0.74 (95% CI, 0.52–1.06; I^2^ = 7.4%, p_H_ = 0.374, **Figure 3**), suggesting no difference on response rate between HBV-HCC and HCV-HCC patients. Although the funnel plot was visually asymmetrical, no publication bias was observed (Egger’s test, p = 0.988, **Supplementary Figure S2A**). According to sensitivity analysis, LAbbe’ plot, and Galbraith plot, there was little heterogeneity among the studies (**Supplementary Figures S2B–D**).

### Impact of Viral Status on HCC Microenvironment

**Figure 4:**
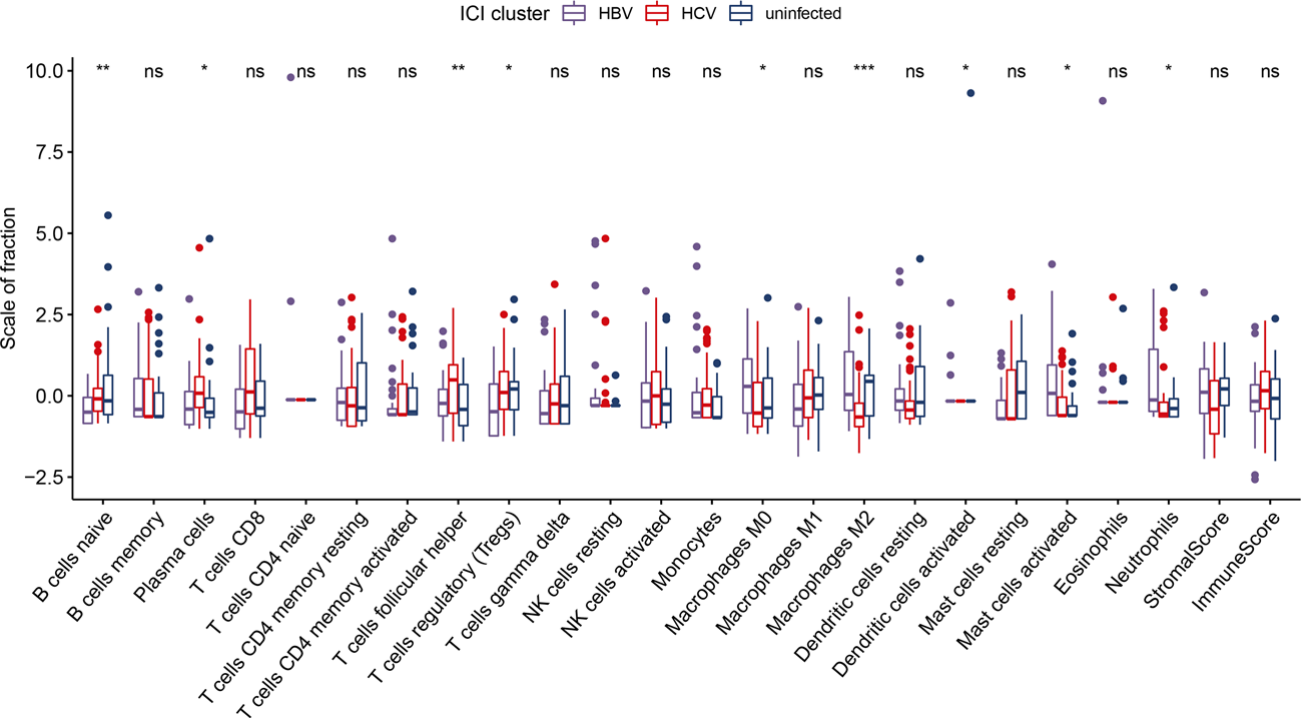
The fraction of tumor-infiltrating immune cells in HCC stratified by etiology. ns, not significant *p < 0.05; **p < 0.01; ***p < 0.001.

We analyzed the immune cell composition of the TME to further depict the global immune infiltration landscape in HCC with different etiology. Among the three subgroups, patients in the HBV-HCC group were characterized by a significantly higher percentage of M0 macrophages, activated dendritic cells, and activated mast cells. The HCV-HCC group was marked by higher percentage of naive B cells, plasma cells, follicular helper T cells, and regulatory T cells. The non-viral HCC group exhibited a significant increase in the infiltration of M2 macrophages (**Figure 4**). The ESTIMATE algorithm was utilized to quantitatively evaluate the TIME in HCC subgroups. The stromal score stands for tumor matrix components. The higher the score, the more the matrix around the tumor, while the immune score is closely related to the degree of immune cell infiltration. Although differences in infiltration level were observed in specific cell types, the immune and stromal scores were generally comparable among etiology groups, suggesting that viral infection had minimal impact on tumor microenvironment remodeling during HCC tumorigenesis (**Figure 4**).

### Publication Bias and Sensitivity Analysis

Egger’s s linear regression test was conducted to examine potential publication bias. No publication bias among included studies were observed (**Table 2**). Besides, funnel plots were generated for visually assessing publication bias (**Supplementary Figures S1A** and **S2A**).

**Table 2:** Egger’s publication bias test.

Outcomes	No. of study	No. of patients	p for bias	95% CI for bias
Virally infected HCC *vs* uninfected HCC	8	1,296	0.080	−0.204, 2.694
HBV-HCC *vs* HCV-HCC	7	771	0.988	−1.739, 1.763

CI, confidence interval; HCC, hepatocellular carcinoma; HBV, hepatitis B virus; HCV, hepatitis C virus.

## Discussion

ICIs have been used in a wide range of previously untreatable malignancies including viral-associated cancers such as HCC and HNSCC (6, 30). Interestingly, it is observed that some patients with specific viral etiology are more likely to benefit from immunotherapies. A previous study reported that the OS almost doubled in HPV-positive HNSCC compared with HPV-negative patients (31). This is partly because of the synthesis of E6 and E7 oncoproteins in HPV-positive patients that make tumor cells extremely detectable to the immune system (32). A recent pan-cancer analysis of over 10,000 samples from 23 cancer types revealed a significantly higher infiltration of B cells, CD4, and CD8 T cells in HPV-positive HNSCC than HPV-negative HNSCC (10). Besides, bioinformatic analysis of TCGA datasets indicated an increased cytolytic activity in EBV-positive stomach cancer and HPV-positive HNSCC, urothelial cancer, and cervical cancer (33). These observations suggest that proteins from oncogenic viruses may act as immunogenic neoantigens and is associated with an elevated immune response.

Unlike other human malignancies, the etiological factors of HCC are well established and vary substantially by geographical regions. HBV infection and aflatoxin exposure are likely the predominant causes in Asia and Africa (34, 35), while HCV, alcoholism, and non-alcoholic fatty liver disease (NAFLD) represent major risk factors in other areas of the world, and the latter has become an emerging risk factor for HCC over the past decade (1, 36). Currently, 3.5% of the global population is chronically infected with HBV, up to 40% of which will eventually develop HCC (34). On the other hand, it is estimated that at least 3.5 million people are currently infected with HCV in the United States, and 20% of liver cancer mortality worldwide is associated with HCV infection (37, 38). Chronic hepatitis, either caused by HBV, HCV, or non-alcoholic steatohepatitis (NASH), leads to an immunosuppressive intrahepatic microenvironment, which is marked by an increased expression of inhibitory receptors on the surface of cytotoxic T cells (39). Blockage of these inhibitory receptors and their legends may, on the one hand, reinvigorate tumor-specific T-cell immunity; on the other hand, restore antiviral intrahepatic T-cell responses.

The efficacy of systematic therapy might be affected by different underlying HCC etiologies, with diverse intrahepatic tumor microenvironments distinctly regulating HCC tumorigenesis and immune response. It is still presently controversial whether these etiological differences of oncogenic mechanisms result in difference in response to HCC immunotherapy (40). Different from other viral-associated cancers, responses to ICI were observed irrespective of etiology in HCC. Early phase trails demonstrated that responses to ICIs were generally consistent across HBV-HCC, HCV-HCC, and nonviral-HCC patients, even though the results were not powered for statistical comparisons due to small subgroups (14, 26–28). In line with a previous study with relatively smaller samples, we observed no significant difference in response between virally infected HCC and nonviral-HCC patients (41). Our further analysis suggested that response rates were similar in HBV- and HCV-infected patients. Distinct from other viral-associated malignancies, we observed no etiological difference in the efficacy of immunotherapy in HCC, which may partly result from the unique biological features of hepatitis virus. HBV and HCV are non-cytopathic compared with other tumor-associated viruses (42, 43). With the unique ability to integrate into the host cell genome, persistent viral replication leads to continuous necroinflammation, fibrosis, and aberrant hepatocyte regeneration. High levels of virus and viral antigens together with the naturally immune suppressive environment of the liver contribute to an immunosuppressive tumor microenvironment. We hypothesized that viral-associated HCC is driven by the necroinflammation and cirrhosis due to persistent viral replication, rather than virus-specific immune responses.

Recently, a study provided some new insights into HCC immunotherapy. In this study, based on results from preclinical tests, the authors indicated that tumor immune surveillance was impaired in NASH-induced HCC because CD8+ T cells helped to induce NASH-HCC, rather than invigorating or executing immune surveillance. The authors further conducted a meta-analysis that showed that NASH-HCC was more refractory to ICIs than viral-HCC (44). However, these findings were based on a retrospective study that included a small population of NASH-associated HCC and different lines of treatment. Therefore, additional studies are warranted to confirm the benefit of ICIs in virally infected HCC patients.

The TME is typically a complex and heterogeneous ecosystem with multiple interacting components. Effector cells including CD8 T cells, NK cells, and suppressive immune cells, such as Tregs, M2 macrophages, and myeloid-derived suppressor cells (MDSCs), are the major components of TME. Immune and immunosuppressive cells are abundant in HCC mellitus, balancing the cancer immunity and regulating immune response (45). Generally, macrophages consist of two polarization states, namely, M1 macrophages and M2 macrophages, which exhibit opposing roles in HCC tumorigenesis. M1 macrophages, also known as classically activated macrophages, exert antitumorigenic roles by releasing toxic intermediates including reactive oxygen species (ROS) and stimulating naive T cells to make a Th1/cytotoxic response (46). In contrast, M2 macrophages are polarized by Th2 cytokines and associated with cancer proliferation, angiogenesis, and ECM remodeling (25). Tregs cells are the subset of CD4+ T cells and characterized by the CD25 marker, which are known to exert immunosuppressive effects, and the presence of Tregs is often correlated with poor prognosis in HCC (47, 48). We found that more Tregs were infiltrated in HCV-HCC than in HBV-HCC, consistent with a previous study that demonstrated that Tregs were significantly higher in HCV related HCC, especially in the recurrence subset (49).

Characterizing the heterogeneous populations of tumor-infiltrating immune cells in TME may help to deepen our understandings of immune response in HCC tumorigenesis, thus boosting the development of effective immunotherapy (50). Computational algorithms including CIBERSORT and ESTIMATE have been widely utilized to accurately calculate the abundance of 22 immune cell types in TME using gene expression matrix (24, 51). It has previously been demonstrated that HCC etiology was not associated with the expression of genes within a Th1/IFN-γ–related immune signature, which may be predictive of immunotherapy response (41). Here, we demonstrated that specific immune cell types demonstrated different infiltration level, even though no significantly difference was observed in response to ICIs among HCC patients with difference etiology. The reason for this disparity is unclear, as TME remodeling during cancer progression is a complex process that involves a variety of sophisticated regulatory mechanisms, which may not only be determined by a specific cell type. On the other hand, the infiltration of immune cells in TME is calculated based on gene expression matrix using imputation algorithm, which may not fully reflect the real state of TME. TME is considered a complex milieu of intervention with multiple interacting components. The 22 immune cell phenotypes are only a tip of the iceberg, and large portions of immune cells that exert distinct functions are still unknown.

The landscape of novel therapeutic agents and combinations towards aHCC are quickly evolving in the past decade. Inspired by the landmark results of the IMbrave150 Phase III trial, immune-based combinations including durvalumab plus tremelimumab, cabozantinib plus atezolizumab, lenvatinib plus pembrolizumab, and nivolumab plus ipilimumab are under assessment, which may further modify the therapeutic scenario in patients with aHCC in the next 5 years (17, 52). The recent ORIENT-32 study also reported an unprecedented benefit for sintilimab plus bevacizumab biosimilar *versus* sorafenib monotherapy in the first-line setting for Chinese patients with aHCC (53). We suggest that further efforts should be oriented towards the identification of potential populations who may benefit from immunotherapy and thus help in guiding individualized treatment. In addition, the clinicopathological features of HCC varies among different patient populations. For instance, patients with HBV-associated HCC in China are characterized by younger age, poor ECOG performance status, and increased risk of distant metastasis compared with patients in western countries (53). Thus, additional studies using the real-world data combined with clinical data from different endemic areas are warranted to evaluate the efficacy and safety of ICI-based immunotherapies.

Several limitations should be acknowledged in the current study. First, the current meta-analysis only encompassed a total of eight studies. In fact, the number of studies reported response to ICIs stratified by etiology was relatively small, which may compromise the credibility of the results. Second, most studies included were early phase studies, and more RCTs were needed for higher level evidence. Third, only ORR was illustrated to define the response to ICI. Unfortunately, few studies included in our meta-analysis differentiate between viral and non-viral HCC when they reported OS or PFS; therefore, our ability to distill responses into viral etiology was limited. ORR is the only reliable endpoint to generate statistically powerful meta-data based on the available data. Forth, we failed to differentiate between alcoholic liver disease and NAFLD or NASH in non-virally infected subgroup due to limited data available. Last, the findings from bioinformatics analysis warrant further experimental validation.

Although previous study had investigated the ORR for PD-1/L1 inhibitors in virally infected and uninfected HCC, the number of articles included was relatively small (41). Besides, it has not been well clarified previously whether there is a difference in response to ICIs between HBV-HCC and HCV-HCC patients. The current study comprehensively investigated the etiology of HCC and the efficacy of ICIs and suggested that viral etiology may not be considered as the selection criteria for patients receiving ICIs. We further took an in-depth view of the immune infiltration pattern and suggested that HCC etiology did not have an apparent effect on TME remodeling, which provide new sight for HCC immunotherapy.

## Author Contributions

ZND and TL conceived and designed the study. SY, YCY, and CY independently screened the full text of selected studies to confirm eligibility, assess quality, and extract data. ZND, YFY, LY, HL analyzed the data. ZND, ZRD, ZC, JH, and TL wrote, reviewed, and/or revised the manuscript. All authors contributed to the article and approved the submitted version.

## Funding

This work was supported by the Taishan Scholars Program for Young Expert of Shandong Province (tsqn20161064), the National Natural Science Foundation of China (81874178 and 82073200), and funds for Independent Cultivation of Innovative Team from Universities in Jinan (Grant No. 2020GXRC023).

## Conflict of Interest

The authors declare that the research was conducted in the absence of any commercial or financial relationships that could be construed as a potential conflict of interest.

## Publisher’s Note

All claims expressed in this article are solely those of the authors and do not necessarily represent those of their affiliated organizations, or those of the publisher, the editors and the reviewers. Any product that may be evaluated in this article, or claim that may be made by its manufacturer, is not guaranteed or endorsed by the publisher.

## References

[1] Sung H Ferlay J Siegel RL Laversanne M Soerjomataram I Jemal A. Global Cancer Statistics 2020: GLOBOCAN Estimates of Incidence and Mortality Worldwide for 36 Cancers in 185 Countries. CA Cancer J Clin (2021) 71(3):209–49. doi: 10.3322/caac.21660 33538338

[2] Wilhelm S Carter C Lynch M Lowinger T Dumas J Smith RA. Discovery and Development of Sorafenib: A Multikinase Inhibitor for Treating Cancer. Nat Rev Drug Discov (2006) 5(10):835–44. doi: 10.1038/nrd2130 17016424

[3] Gordan JD Kennedy EB Abou-Alfa GK Beg MS Brower ST Gade TP. Systemic Therapy for Advanced Hepatocellular Carcinoma: ASCO Guideline. J Clin Oncol (2020) 38(36):4317–45. doi: 10.1200/JCO.20.02672 33197225

[4] Rizzo A Dadduzio V Ricci AD Massari F Di Federico A Gadaleta-Caldarola G. Lenvatinib Plus Pembrolizumab: The Next Frontier for the Treatment of Hepatocellular Carcinoma? Expert Opin Investig Drugs (2021) 1–8. doi: 10.1080/13543784.2021.1948532 34167433

[5] Finn RS Ikeda M Zhu AX Sung MW Baron AD Kudo M. Phase Ib Study of Lenvatinib Plus Pembrolizumab in Patients With Unresectable Hepatocellular Carcinoma. J Clin Oncol (2020) 38(26):2960–70. doi: 10.1200/JCO.20.00808 PMC7479760 32716739

[6] Finn RS Qin S Ikeda M Galle PR Ducreux M Kim TY. Atezolizumab Plus Bevacizumab in Unresectable Hepatocellular Carcinoma. N Engl J Med (2020) 382(20):1894–905. doi: 10.1056/NEJMoa1915745 32402160

[7] de Martel C Georges D Bray F Ferlay J Clifford GM. Global Burden of Cancer Attributable to Infections in 2018: A Worldwide Incidence Analysis. Lancet Glob Health (2020) 8(2):e180–90. doi: 10.1016/S2214-109X(19)30488-7 31862245

[8] Ferris RL Blumenschein G Jr Fayette J Guigay J Colevas AD Licitra L. Nivolumab for Recurrent Squamous-Cell Carcinoma of the Head and Neck. N Engl J Med (2016) 375(19):1856–67. doi: 10.1056/NEJMoa1602252 PMC5564292 27718784

[9] Seiwert TY Burtness B Mehra R Weiss J Berger R Eder JP. Safety and Clinical Activity of Pembrolizumab for Treatment of Recurrent or Metastatic Squamous Cell Carcinoma of the Head and Neck (KEYNOTE-012): An Open-Label, Multicentre, Phase 1b Trial. Lancet Oncol (2016) 17(7):956–65. doi: 10.1016/S1470-2045(16)30066-3 27247226

[10] Li B Severson E Pignon JC Zhao H Li T Novak J. Comprehensive Analyses of Tumor Immunity: Implications for Cancer Immunotherapy. Genome Biol (2016) 17(1):174. doi: 10.1186/s13059-016-1028-7 27549193 PMC4993001

[11] Park JW Chen M Colombo M Roberts LR Schwartz M Chen PJ. Global Patterns of Hepatocellular Carcinoma Management From Diagnosis to Death: The BRIDGE Study. Liver Int (2015) 35(9):2155–66. doi: 10.1111/liv.12818 PMC4691343 25752327

[12] Ali A Abdel-Hafiz H Suhail M Al-Mars A Zakaria MK Fatima K. Hepatitis B Virus, HBx Mutants and Their Role in Hepatocellular Carcinoma. World J Gastroenterol (2014) 20(30):10238–48. doi: 10.3748/wjg.v20.i30.10238 PMC4130832 25132741

[13] Yau T Kang YK Kim TY El-Khoueiry AB Santoro A Sangro B. Efficacy and Safety of Nivolumab Plus Ipilimumab in Patients With Advanced Hepatocellular Carcinoma Previously Treated With Sorafenib: The CheckMate 040 Randomized Clinical Trial. JAMA Oncol (2020) 6(11):e204564. doi: 10.1001/jamaoncol.2020.4564 33001135 PMC7530824

[14] Feun LG Li YY Wu C Wangpaichitr M Jones PD Richman SP. Phase 2 Study of Pembrolizumab and Circulating Biomarkers to Predict Anticancer Response in Advanced, Unresectable Hepatocellular Carcinoma. Cancer (2019) 125(20):3603–14. doi: 10.1002/cncr.32339 PMC7592647 31251403

[15] Li X Liu R Su X Pan Y Han X Shao C. Harnessing Tumor-Associated Macrophages as Aids for Cancer Immunotherapy. Mol Cancer (2019) 18(1):177. doi: 10.1186/s12943-019-1102-3 31805946 PMC6894344

[16] Kelley RK Sangro B Harris W Ikeda M Okusaka T Kang YK. Safety, Efficacy, and Pharmacodynamics of Tremelimumab Plus Durvalumab for Patients With Unresectable Hepatocellular Carcinoma: Randomized Expansion of a Phase I/II Study. J Clin Oncol (2021) 39(27):2991–3001. doi: 10.1200/JCO.20.03555 34292792 PMC8445563

[17] Rizzo A Ricci AD Brandi G. Atezolizumab in Advanced Hepatocellular Carcinoma: Good Things Come to Those Who Wait. Immunotherapy (2021) 13(8):637–44. doi: 10.2217/imt-2021-0026 33820447

[18] Page MJ Moher D Bossuyt PM Boutron I Hoffmann TC Mulrow CD. PRISMA 2020 Explanation and Elaboration: Updated Guidance and Exemplars for Reporting Systematic Reviews. BMJ (2021) 372:n160. doi: 10.1136/bmj.n160 33781993 PMC8005925

[19] Guo B Moga C Harstall C Schopflocher D. A Principal Component Analysis Is Conducted for a Case Series Quality Appraisal Checklist. J Clin Epidemiol (2016) 69:199–207.e2. doi: 10.1016/j.jclinepi.2015.07.010 26307459

[20] Cancer Genome Atlas Research Network. Comprehensive and Integrative Genomic Characterization of Hepatocellular Carcinoma. Cell (2017) 169(7):1327–41.e23. doi: 10.1016/j.cell.2017.05.046 28622513 PMC5680778

[21] Wagner GP Kin K Lynch VJ. Measurement of mRNA Abundance Using RNA-Seq Data: RPKM Measure Is Inconsistent Among Samples. Theory Biosci (2012) 131(4):281–5. doi: 10.1007/s12064-012-0162-3 22872506

[22] Hänzelmann S Castelo R Guinney J. GSVA: Gene Set Variation Analysis for Microarray and RNA-Seq Data. BMC Bioinf (2013) 14:7. doi: 10.1186/1471-2105-14-7 PMC3618321 23323831

[23] Bindea G Mlecnik B Tosolini M Kirilovsky A Waldner M Obenauf AC. Spatiotemporal Dynamics of Intratumoral Immune Cells Reveal the Immune Landscape in Human Cancer. Immunity (2013) 39(4):782–95. doi: 10.1016/j.immuni.2013.10.003 24138885

[24] Yoshihara K Shahmoradgoli M Martínez E Vegesna R Kim H Torres-Garcia W. Inferring Tumour Purity and Stromal and Immune Cell Admixture From Expression Data. Nat Commun (2013) 4:2612. doi: 10.1038/ncomms3612 24113773 PMC3826632

[25] Tahmasebi Birgani M Carloni V. Tumor Microenvironment, A Paradigm in Hepatocellular Carcinoma Progression and Therapy. Int J Mol Sci (2017) 18(2):405. doi: 10.3390/ijms18020405 PMC5343939 28216578

[26] El-Khoueiry AB Sangro B Yau T Crocenzi TS Kudo M Hsu C. Nivolumab in Patients With Advanced Hepatocellular Carcinoma (CheckMate 040): An Open-Label, Non-Comparative, Phase 1/2 Dose Escalation and Expansion Trial. Lancet (2017) 389(10088):2492–502. doi: 10.1016/S0140-6736(17)31046-2 PMC7539326 28434648

[27] Lee MS Ryoo BY Hsu CH Numata K Stein S Verret W. Atezolizumab With or Without Bevacizumab in Unresectable Hepatocellular Carcinoma (GO30140): An Open-Label, Multicentre, Phase 1b Study. Lancet Oncol (2020) 21(6):808–20. doi: 10.1016/S1470-2045(20)30156-X 32502443

[28] Zhu AX Finn RS Edeline J Cattan S Ogasawara S Palmer D. Pembrolizumab in Patients With Advanced Hepatocellular Carcinoma Previously Treated With Sorafenib (KEYNOTE-224): A Non-Randomised, Open-Label Phase 2 Trial. Lancet Oncol (2018) 19(7):940–52. doi: 10.1016/S1470-2045(18)30351-6 29875066

[29] Wainberg ZA Segal NH Jaeger D Lee K-H Marshall J Antonia SJ. Safety and Clinical Activity of Durvalumab Monotherapy in Patients With Hepatocellular Carcinoma (HCC). J Clin Oncol (2017) 35(15_suppl):4071. doi: 10.1200/JCO.2017.35.15_suppl.4071

[30] Mandal R Şenbabaoğlu Y Desrichard A Havel JJ Dalin MG Riaz N. The Head and Neck Cancer Immune Landscape and Its Immunotherapeutic Implications. JCI Insight (2016) 1(17):e89829. doi: 10.1172/jci.insight.89829 27777979 PMC5070962

[31] Galvis MM Borges GA Oliveira TB Toledo IP Castilho RM Guerra ENS. Immunotherapy Improves Efficacy and Safety of Patients With HPV Positive and Negative Head and Neck Cancer: A Systematic Review and Meta-Analysis. Crit Rev Oncol Hematol (2020) 150:102966. doi: 10.1016/j.critrevonc.2020.102966 32371338

[32] Welters MJP Ma W Santegoets S Goedemans R Ehsan I Jordanova ES. Intratumoral HPV16-Specific T Cells Constitute a Type I-Oriented Tumor Microenvironment to Improve Survival in HPV16-Driven Oropharyngeal Cancer. Clin Cancer Res (2018) 24(3):634–47. doi: 10.1158/1078-0432.CCR-17-2140 29018052

[33] Rooney MS Shukla SA Wu CJ Getz G Hacohen N. Molecular and Genetic Properties of Tumors Associated With Local Immune Cytolytic Activity. Cell (2015) 160(1-2):48–61. doi: 10.1016/j.cell.2014.12.033 25594174 PMC4856474

[34] Yuen MF Chen DS Dusheiko GM Janssen HLA Lau DTY Locarnini SA. Hepatitis B Virus Infection. Nat Rev Dis Primers (2018) 4:18035. doi: 10.1038/nrdp.2018.35 29877316

[35] Villanueva A. Hepatocellular Carcinoma. N Engl J Med (2019) 380(15):1450–62. doi: 10.1056/NEJMra1713263 30970190

[36] Anstee QM Reeves HL Kotsiliti E Govaere O Heikenwalder M. From NASH to HCC: Current Concepts and Future Challenges. Nat Rev Gastroenterol Hepatol (2019) 16(7):411–28. doi: 10.1038/s41575-019-0145-7 31028350

[37] Edlin BR Eckhardt BJ Shu MA Holmberg SD Swan T. Toward a More Accurate Estimate of the Prevalence of Hepatitis C in the United States. Hepatology (2015) 62(5):1353–63. doi: 10.1002/hep.27978 PMC4751870 26171595

[38] Plummer M de Martel C Vignat J Ferlay J Bray F Franceschi S. Global Burden of Cancers Attributable to Infections in 2012: A Synthetic Analysis. Lancet Glob Health (2016) 4(9):e609–16. doi: 10.1016/S2214-109X(16)30143-7 27470177

[39] Riley RS June CH Langer R Mitchell MJ. Delivery Technologies for Cancer Immunotherapy. Nat Rev Drug Discovery (2019) 18(3):175–96. doi: 10.1038/s41573-018-0006-z PMC6410566 30622344

[40] Bruix J Raoul JL Sherman M Mazzaferro V Bolondi L Craxi A. Efficacy and Safety of Sorafenib in Patients With Advanced Hepatocellular Carcinoma: Subanalyses of a Phase III Trial. J Hepatol (2012) 57(4):821–9. doi: 10.1016/j.jhep.2012.06.014 PMC12261288 22727733

[41] Ho WJ Danilova L Lim SJ Verma R Xavier S Leatherman JM. Viral Status, Immune Microenvironment and Immunological Response to Checkpoint Inhibitors in Hepatocellular Carcinoma. J Immunother Cancer (2020) 8(1):e000394. doi: 10.1136/jitc-2019-000394 32303615 PMC7204805

[42] Akbar SK Horiike N Onji M. Prognostic Importance of Antigen-Presenting Dendritic Cells During Vaccine Therapy in a Murine Hepatitis B Virus Carrier. Immunology (1999) 96(1):98–108. doi: 10.1046/j.1365-2567.1999.00668.x 10233683 PMC2326722

[43] Alter MJ Kruszon-Moran D Nainan OV McQuillan GM Gao F Moyer LA. The Prevalence of Hepatitis C Virus Infection in the United States, 1988 Through 1994. N Engl J Med (1999) 341(8):556–62. doi: 10.1056/NEJM199908193410802 10451460

[44] Pfister D Núñez NG Pinyol R Govaere O Pinter M Szydlowska M. NASH Limits Anti-Tumour Surveillance in Immunotherapy-Treated HCC. Nature (2021) 592(7854):450–6. doi: 10.1038/s41586-021-03362-0 PMC8046670 33762733

[45] Bian J Lin J Long J Yang X Yang X Lu X. T Lymphocytes in Hepatocellular Carcinoma Immune Microenvironment: Insights Into Human Immunology and Immunotherapy. Am J Cancer Res (2020) 10(12):4585–606. PMC7783774 33415021

[46] Pei Y Yeo Y. Drug Delivery to Macrophages: Challenges and Opportunities. J Control Release (2016) 240:202–11. doi: 10.1016/j.jconrel.2015.12.014 26686082

[47] Fu J Xu D Liu Z Shi M Zhao P Fu B. Increased Regulatory T Cells Correlate With CD8 T-Cell Impairment and Poor Survival in Hepatocellular Carcinoma Patients. Gastroenterology (2007) 132(7):2328–39. doi: 10.1053/j.gastro.2007.03.102 17570208

[48] Peltanova B Raudenska M Masarik M. Effect of Tumor Microenvironment on Pathogenesis of the Head and Neck Squamous Cell Carcinoma: A Systematic Review. Mol Cancer (2019) 18(1):63. doi: 10.1186/s12943-019-0983-5 30927923 PMC6441173

[49] Yoshizawa K Abe H Kubo Y Kitahara T Aizawa R Matsuoka M. Expansion of CD4(+)CD25(+)FoxP3(+) Regulatory T Cells in Hepatitis C Virus-Related Chronic Hepatitis, Cirrhosis and Hepatocellular Carcinoma. Hepatol Res (2010) 40(2):179–87. doi: 10.1111/j.1872-034X.2009.00587.x 20070404

[50] Roderburg C Wree A Demir M Schmelzle M Tacke F. The Role of the Innate Immune System in the Development and Treatment of Hepatocellular Carcinoma. Hepatic Oncol (2020) 7(1):Hep17. doi: 10.2217/hep-2019-0007 PMC7137177 32273975

[51] Newman AM Liu CL Green MR Gentles AJ Feng W Xu Y. Robust Enumeration of Cell Subsets From Tissue Expression Profiles. Nat Methods (2015) 12(5):453–7. doi: 10.1038/nmeth.3337 PMC4739640 25822800

[52] Kelley RK WO J Hazra S Benzaghou F Yau T Cheng AL. Cabozantinib in Combination With Atezolizumab *Versus* Sorafenib in Treatment-Naive Advanced Hepatocellular Carcinoma: COSMIC-312 Phase III Study Design. Future Oncol (2020) 16(21):1525–36. doi: 10.2217/fon-2020-0283 32491932

[53] Ren Z Xu J Bai Y Xu A Cang S Du C. Sintilimab Plus a Bevacizumab Biosimilar (IBI305) *Versus* Sorafenib in Unresectable Hepatocellular Carcinoma (ORIENT-32): A Randomised, Open-Label, Phase 2-3 Study. Lancet Oncol (2021) 22(7):977–90. doi: 10.1016/S1470-2045(21)00252-7 34143971

