# Supplementary material for: Viral Status and Efficacy of Immunotherapy in Hepatocellular Carcinoma: A Systematic Review With Meta-Analysis
Source: Front Immunol. 2021 Sep 29;12:733530. doi: 10.3389/fimmu.2021.733530 (PMC8511422; doi:10.3389/fimmu.2021.733530)
Supplement: Supplementary file 1 [file DataSheet_1.docx]

Supplementary Material

# Supplementary Table 1

Detailed search strategy was as follows: (carcinoma, hepatocellular) AND (((immunotherapy) OR immune checkpoint inhibitors) OR (((((((nivolumab) OR pembrolizumab) OR atezolizumab) OR avelumab) OR camrelizumab) OR SHR-1210) OR durvalumab) OR ((((programmed cell death 1 receptor) OR programmed cell death-1) OR PD-1 receptor) OR CD279 antigen) OR ((((B7-H1 antigen) OR programmed death ligand 1) OR PD-L1 protein) OR CD274 antigen)).

# Supplementary Figures


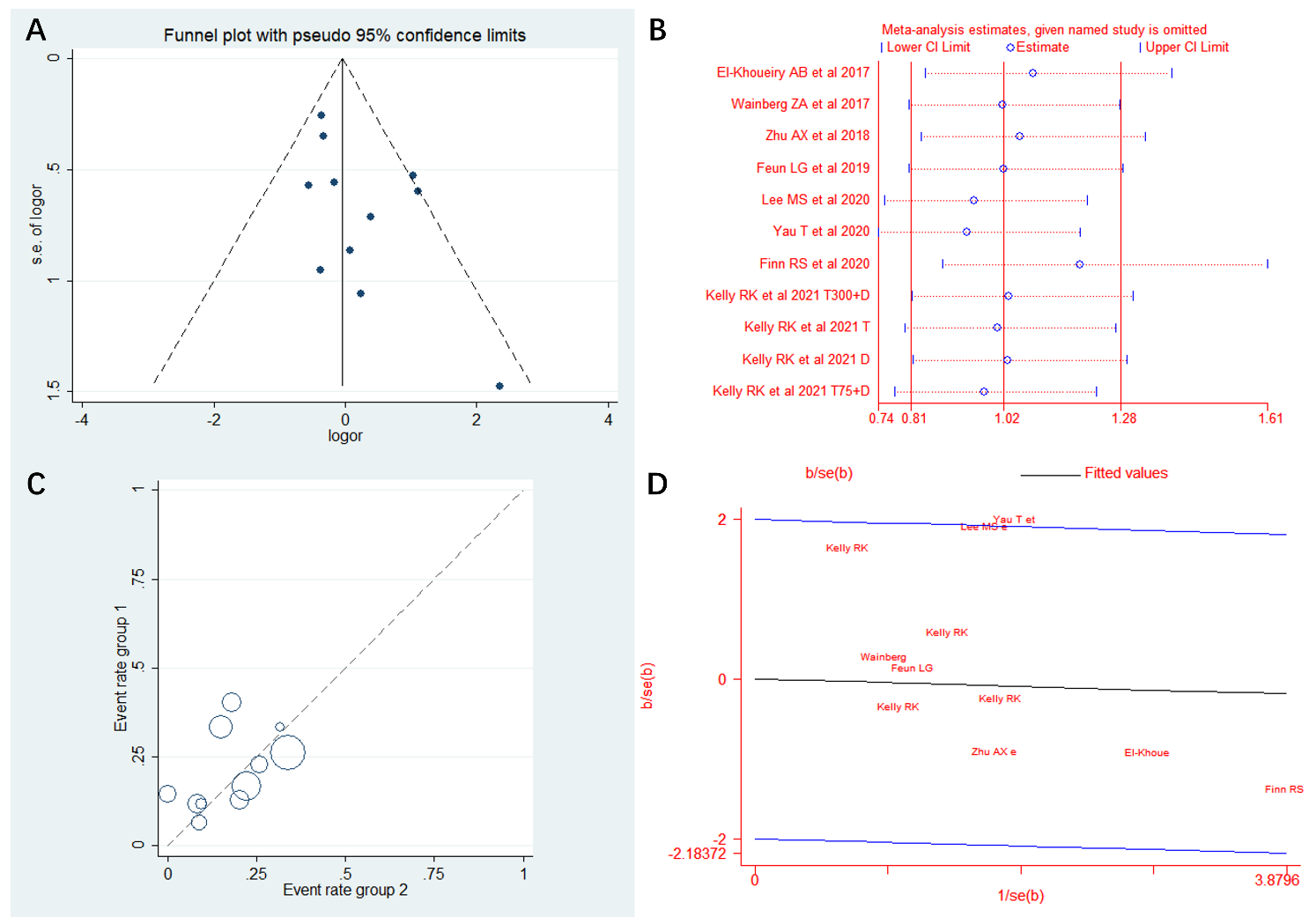


Supplementary Figure. 1

Figure. S1 (A) Invert funnel plot. (B) Impact of single study on pooled odds ratio. (C) L'Abbé plot was generated to visually evaluate heterogeneity among studies. (D) Galbraith plot was conducted to investigate the potential source of heterogeneity.


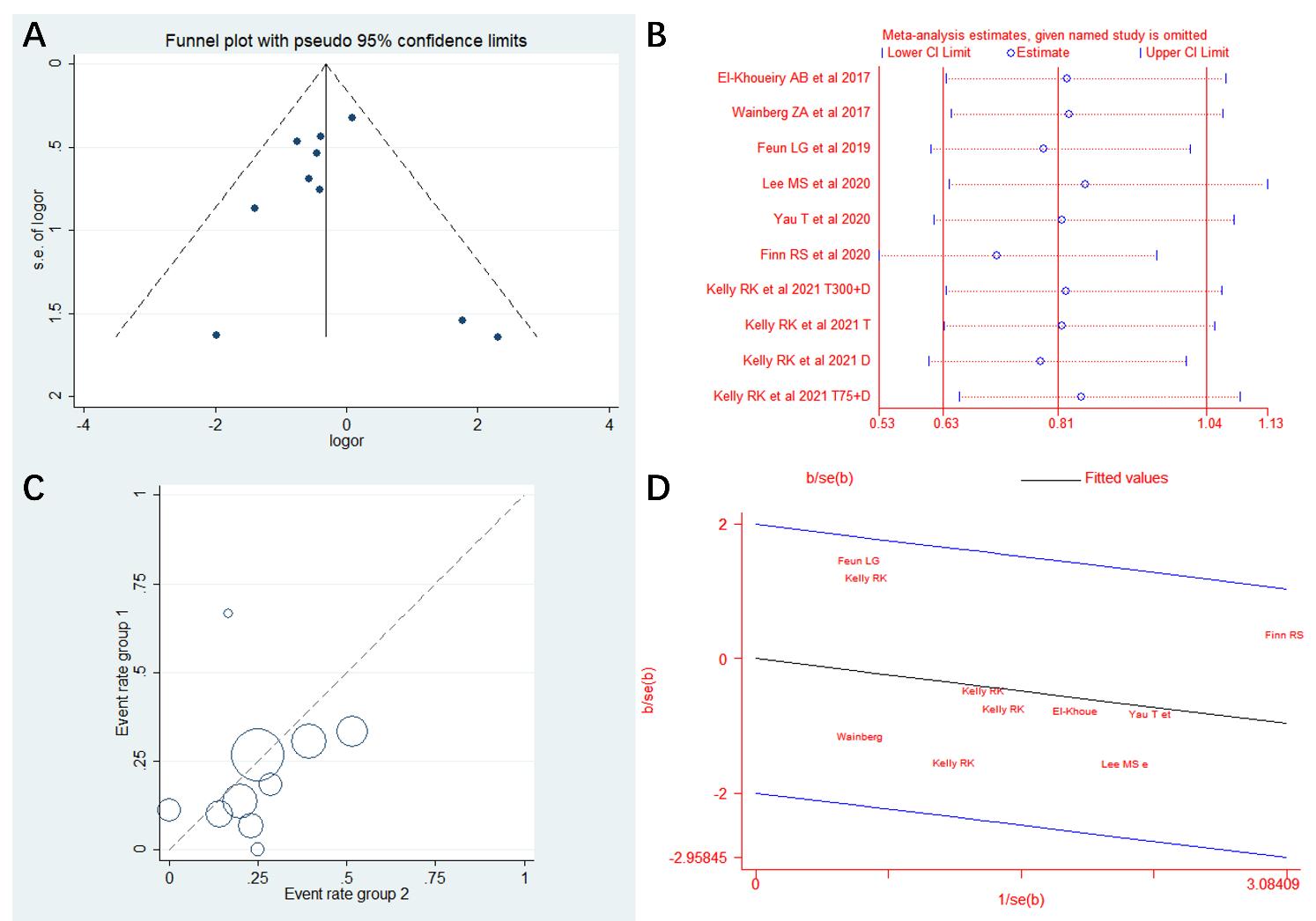


Supplementary Figure. 2

Figure. S2 (A) Invert funnel plot. (B) Impact of single study on pooled odds ratio. (C) L'Abbé plot was generated to visually evaluate heterogeneity among studies. (D) Galbraith plot was conducted to investigate the potential source of heterogeneity.
